# Estimating the contribution of the gut to plasma viral load in early SIV infection

**DOI:** 10.1186/1742-4690-10-105

**Published:** 2013-10-14

**Authors:** Janka Petravic, Thomas H Vanderford, Guido Silvestri, Miles Davenport

**Affiliations:** 1 Complex Systems in Biology Group, Centre for Vascular Research, University of New South Wales, Kensington, NSW 2052, Australia; 2 Emory University School of Medicine and Yerkes National Primate Research Center, Emory University, Atlanta, GA, USA

**Keywords:** Simian immunodeficiency virus infection, Gut, Immune escape

## Abstract

**Background:**

There is significant debate about whether the gut plays a major role in viral replication and pathology in HIV infection. Here we aimed to estimate the contribution of the gut to the total virus observed in plasma, by comparing the frequency of different viral mutants in plasma and gut in SIV infection.

**Results:**

We found that the maximum contribution of gut to plasma viral load estimated from rectal biopsy at day 28 post-infection had a median of 10%. The estimated values for individual animals ranged from nearly 100% to <3% in 4/14 animals. Importantly, these are maximum estimates, so that a value of 90%, for example, means that the real contribution may be anything between 0 and 90%, just not higher than 90%.

We also studied the contribution of gut at the peak of plasma viral load (day 14). However, since there was very little escape in most animals at this time point, we could only estimate the maximal contribution of gut in 4 animals, in two of which it was <15%.

**Conclusions:**

The role of the gut in HIV is a controversial area, with many suggesting that it plays a dominant role in driving early infection. Our analysis suggests that, at least by day 28 post-infection, the gut is not contributing greatly to the plasma viral load.

## Background

In the first weeks of human immunodeficiency virus (HIV) infection in humans and simian immunodeficiency virus (SIV) infection in rhesus macaques, the loss of CD4+ T lymphocytes in peripheral blood is moderate and transient. In contrast, there is a rapid and nearly complete depletion of intestinal CD4+ T cells within first weeks of infection both in SIV-infected macaques [1-4] and HIV-infected humans [5], which persists during the course of disease. This has been attributed to the very high frequency of the main targets for HIV infection (CD4+ T cells expressing the CCR5 co-receptor) in gut mucosa [6]. It is also often proposed that the gastrointestinal tract is the largest lymphoid organ in the body, containing more than half of the total body T-cells [7,8]. In addition, a high proportion of cells in the lamina propria are CCR5^+^, and thus highly susceptible to infection. This has led to a widely accepted conclusion that the infection of gut CD4+ T cells is the origin of the initial burst of viremia, generating the peak viral load in plasma, and a major contributor to viral production during infection [1,4,9]. Both ideas have recently been disputed. The belief that the largest proportion of CD4+ T cells in the body resides in the gut has been challenged by studies of the post-mortem lymphocyte numbers in mammalian organs [10]. We have shown that it is also improbable that the gut is the main source of the virus at the peak of viremia, since it is already heavily depleted of CD4+ T cells days before the peak [11], so that the observed dynamics of CD4+ T cell depletion is more consistent with the gut being a smaller compartment comprising CD4+ T cells that are highly susceptible to infection [11].

In a recent study of SIV_mac239_-infected rhesus macaques, Vanderford *et al.*[12] followed the appearance of viral escape mutants (EM) in plasma, lymph nodes (LN), rectal biopsy (RB) and peripheral blood mononuclear cells (PBMC), and found evidence that immune pressure in lymph nodes was the most effective in selecting SIV escape variants that later dominated the infection in all tissues. Here we use the data on the content of wild-type (WT) strain in plasma virus and infected cells in different tissues from this study to estimate the maximal contribution of the gut to the plasma viral load during early infection.

## Results and discussion

**Figure 1:**
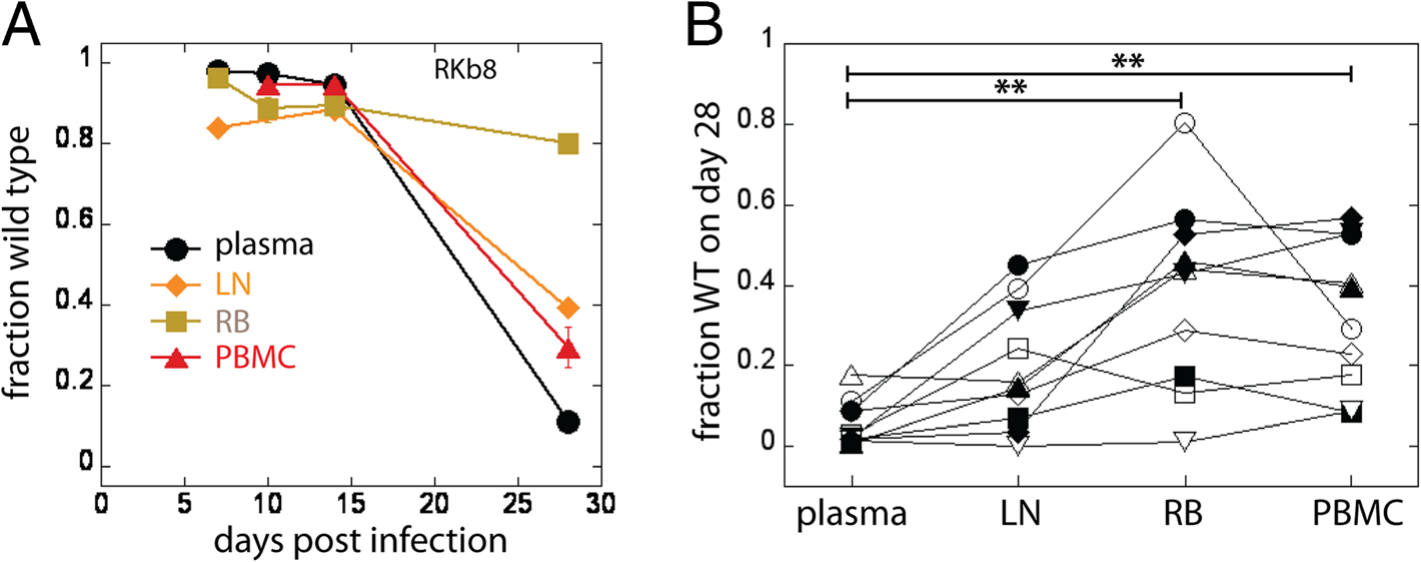
**Viral escape in plasma and tissues. A**. Escape in plasma, lymph nodes, PBMC and rectal biopsy of one animal (error bars represent confidence intervals of a proportion); **B**. Viral escape in the gut and blood lags behind escape in plasma (ie: retaining a higher proportion of WT virus), but not in lymph nodes (Friedman test with Dunn’s multiple comparison post-test). Fractions WT belonging to the same animal in different compartments in B are represented by the same symbol.

We used a modeling approach to estimate the maximal contribution of the gut to plasma viral load. By comparing fractions of WT in plasma and in tissue-infected cells (LN, RB and PBMC) 28 days post infection (approximately 2 weeks post peak), we were able to estimate the largest possible contribution of the virus generated by the infected cells in the gut to plasma viral load. In its simplest form, if the viral DNA in gut is 100% WT, but the virus in plasma is only 10% WT virus, then the gut can contribute at most 10% of the plasma virus. Most samples had between 100 and 9000 sequences, but some (4 LN samples and one RB sample) had only 1–12 sequences. For the multiple comparison test we only used the data on the samples with >100 sequences, so that for this test we only used the data belonging to10 animals. We found that the fraction of WT virus in rectal biopsies and in blood was significantly higher than in plasma, while the difference in WT content between plasma and lymph nodes was not significant (Figure 1; *p =* 0.0018, Friedman test with Dunn’s multiple comparison post-test).

**Figure 2:**
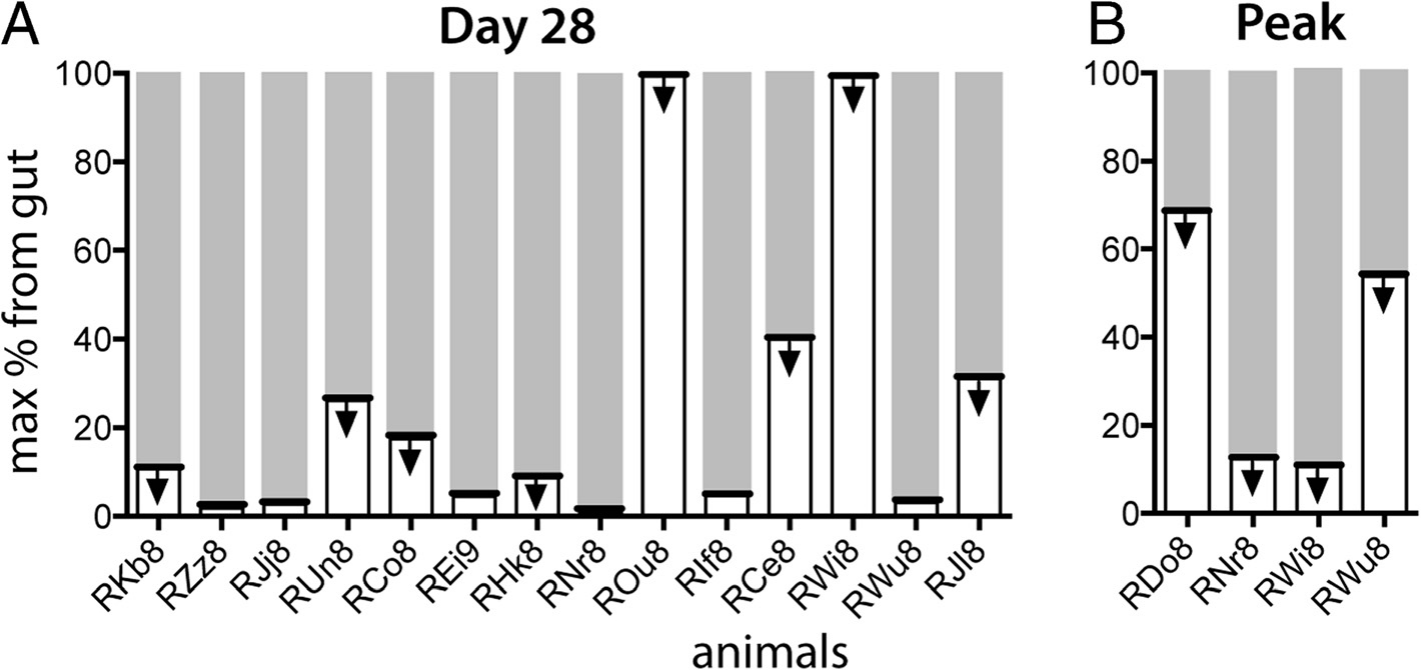
**The highest possible contribution of gut to viral load. A**. On day 28 (white part of the bar), estimated from the difference in WT content in plasma and rectal biopsy; **B**. Analysis of the proportion of WT virus at peak viral load also suggests a low contribution of virus from the gut in the few animals that have escape at this time. The arrows indicate that all estimates are of the maximum contribution of the gut, and the data are equally compatible with any lower figure.

We estimate the highest possible contribution of the gut to the plasma viral load for each sample from the difference in the WT fraction in plasma and in rectal biopsy for each animal. For this estimate we used the data from all 14 animals that had more than 100 sequences in RB samples on day 28. In making this estimate we ignored the data from other tissues (LN and PBMC) because we did not know which other anatomical compartments (for which we have no samples) may be contributing, and how much. When the fraction of WT is higher in RB than in plasma, the gut’s contribution to plasma virus is the highest possible if all WT virus in plasma comes exclusively from the gut. In this case, the fraction of plasma viral load that was produced in the gut would be at most

(1) $$C_{\text{max}}=f_{\text{PL}}/f_{\text{RB}}^{\text{cells}}$$

where *f*_*PL*_ and $f_{\text{RB}}^{\text{cells}}$ are fractions of WT in plasma and rectal biopsy respectively. However, for two animals (ROu8 and RWi8) the WT fraction in RB was very close to, but lower than in plasma. For these two animals, the highest possible fraction of virus that could have come from the gut would be

(2) $$C_{\text{max}}=\left(1-f_{\mathrm{PL}}\right)/\left(1-f_{\text{RB}}^{\text{cells}}\right)$$

when all other tissues were contributing only WT. The highest possible gut contribution to plasma viral load for each animal is shown in Figure 2.

Since this method can only estimate the maximal contribution of gut to plasma viral load, high estimates of this fraction are not very informative. That is, if gut is 100% WT and plasma is 100% WT (ie: no escape has occurred), then we would estimate the maximal contribution is 100%. Similarly, if escape has only proceeded to 50% WT in plasma, we would estimate the maximal gut contribution as 50%. In each case, the contribution of the gut could be any number lower than this (or even zero), and still be compatible with the data. Therefore, although the mean maximal possible gut contribution to plasma is 25% (median 9.8%), the actual contribution must simply be less than this. Since we observe that 7/14 animals have a gut contribution of <10% at day 28 (and 4/14 ≤3%), we suspect that the real contribution is likely substantially less than 10%.

**Figure 3:**
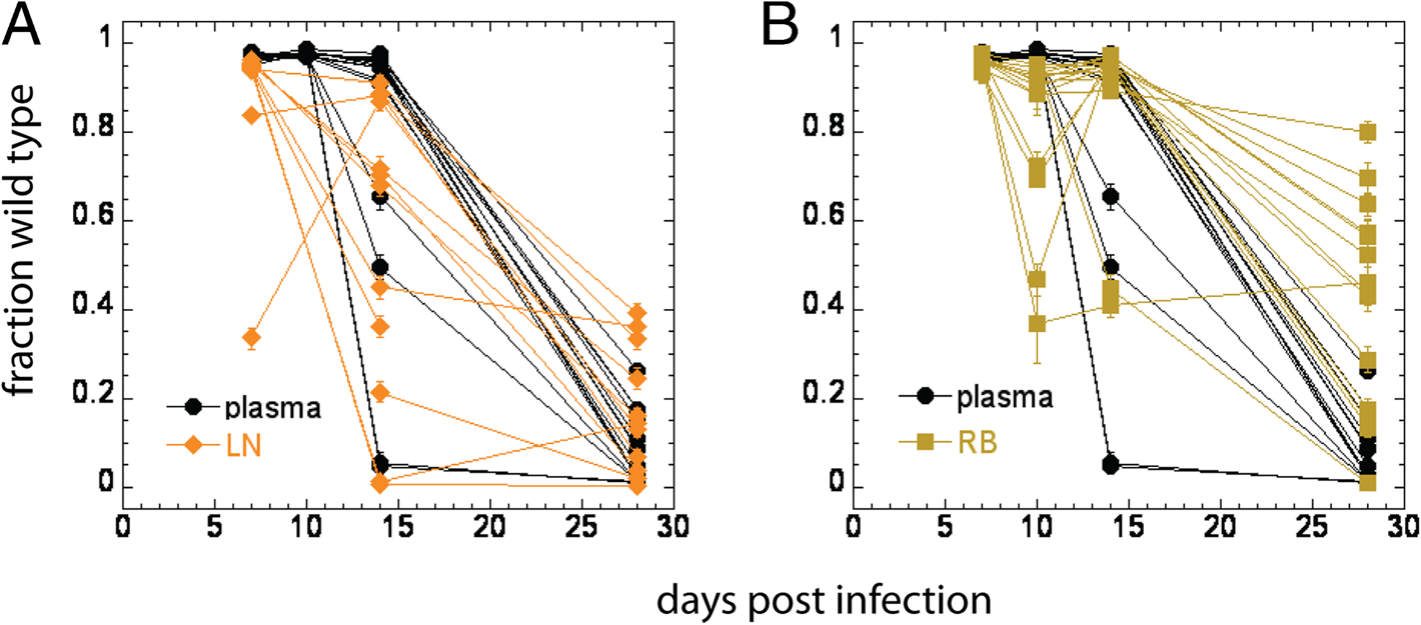
**Longitudinal data on escape in plasma, lymph nodes and rectal biopsy up to day 28. A**. Escape in the lymph nodes precedes escape in plasma from day 14; **B**. In rectal biopsies escape on average lags after plasma after peak viral load on day 28. Error bars represent confidence intervals of a proportion.

We also analyzed the maximal contribution of gut to the plasma viral load at day 14 (the peak of infection). This estimate is uninformative in most animals, since escape has not occurred and both plasma virus and gut DNA virus is close to 100% WT (Additional file 1: Figure S1). However, in four animals where escape had occurred in plasma, we estimated that the maximal contribution of the gut was < 15% for at least two of these animals, suggesting again that the real value of the gut contribution may be very small. This is consistent with our previous work in which we showed that the gut is largely depleted by the time of peak infection [11]. We note in addition that in a few animals the gut showed a brief period of early escape observed on day 10 (Figure 3B). While the mechanisms of this are unclear, the fact that the gut was observed to contain a significant proportion of EM virus, while the plasma remained close to 100% WT virus reinforces the conclusion that also at this early period the gut is unlikely to be a major contributor to plasma viral load.

One caveat to our analysis is that in estimating the contribution of the gut to plasma viral load using Eq.1, we implicitly assumed that the cells infected with WT and EM virus produce free virus at the same average rates. However, WT and infected cells may produce different levels of plasma virus if, for example, either (i) EM virus has a significant fitness cost (so more WT virus than EM virus is produced per infected cell), or (ii) WT viral production is suppressed by WT-specific immune responses (so less WT virus is produced per infected cell). In the case of a fitness cost of escape, this would only decrease the potential contribution of the gut. However, in the second case, this would mean that, for example, if infected cells in gut were 50:50 WT:EM, but WT infected cells produced less virus, we would see less WT in plasma. In this case Eq.1 would underestimate the contribution of gut. If the average production of WT virions *p*_*W*_ is different from the average production of EM strain *p*_*E*_, we have to estimate the highest possible gut contribution to plasma virus *C*_max_ from the expression:

(3) $$C_{\text{max}}=f_{\mathrm{PL}}\left[1+\frac{p_{E}}{p_{W}}\left(\frac{1}{f_{\text{RB}}^{\text{cells}}}-1\right)\right]$$

The derivation of Eq.3 is presented in the Additional file 2.

Simulation of infection and escape where WT virus production is suppressed (see Additional file 3 for the description of the model and Additional file 4 for modeling results) shows that, if production of WT is preferentially suppressed by the immune response, escape in all tissues should always lag after escape in plasma. In this case it is in principle possible that loss of WT is the slowest in the largest compartment, but only if it has much higher infectivity or slower infected cell death rate of infected cells than the other tissues. However, even under this scenario, we expect that escape in plasma should be faster than in all tissues.

The dynamics of escape in lymph nodes and in rectal biopsies is shown in Figure 3. On average, higher levels of escape mutants are seen in LN than plasma from day 14 (Figure 3A), while escape in the gut is on average slower than plasma (Figure 3B). Escape in lymph nodes considerably precedes plasma in some animals (Additional file 4: Figure S6). If escape is occurring due to suppression of WT virus production by CD8+ T cells, it is not possible to see escape in one tissue preceding plasma virus escape and another lagging behind (see Additional file 4: Figure S3 to S5). In addition, no evidence exists for lower virus production by WT-infected cells in vivo, so we do not believe that suppression of viral production is the dominant mechanism of escape.

However, on day 28 the distinction is not this clear, but the dynamics of escape in the gut lag significantly behind escape in plasma, while the difference in the WT level between LN and plasma is not statistically significant, although there is also a (statistically insignificant) trend of a lag after plasma. Maximum estimates of the contribution of LN to plasma on day 28 are higher than for the gut in 9 out of 10 animals for which we have data available, although the median is only 27% (compared to the median maximum contribution of 10% by the gut). This would suggest that LN do not, by themselves, contribute the largest part of the virus in plasma on day 28, but that plasma virus is composed of contributions of different tissues (not all sampled in this study) of which the contribution of the gut is but a small part.

## Conclusions

Although the gastrointestinal tract is often thought of as a large site of susceptible CD4+ T cells and an important site of HIV/SIV infection, this study indicates that it is at best a minor contributor to plasma viral load in early infection. Since our method estimates the maximum contribution of virus produced in RB to plasma virus, we cannot estimate the actual contribution, which could be very low. However, if we assume that the general dynamics of infection in different tissues is similar among all animals, although the dynamics of escape may be much more variable, then the lower estimates of the maximal contribution would be more informative of the actual fraction of virus generated in the gut.

Our study is only able to assess the contribution of the gut to the plasma virus. It is possible that the gut is a large overall producer of virus, but that most of the virus produced in the gut either stays in the gut or is filtered by other tissues on its way to plasma. In this case gut could still be the major producer of the total body virus, but not a major contributor to plasma virus.

Our modeling is based upon an implicit assumption that virus produced in tissues migrates extremely rapidly to plasma. If there were a long delay from virus production in tissues until it reaches plasma, this would affect our analysis. However, studies of viral kinetics under therapy show a very rapid drop in viral loads after initiation of therapy, suggesting that any delays between viral production in tissues and reaching plasma must be very small [13-15].

In our model, we are treating the compartments as if they were isolated. However, there may be mixing of infected cells circulating among compartments. For example, the similarity between PBMC and gut could be explained by PBMC contributing a large proportion of cells obtained in the gut biopsies. Similarly, if a large proportion of PBMC were recycling from the gut, this could also explain the apparent similarity between the gut and PBMC. We do not address these possibilities here, nor are we speculating if the infected cells present in the gut were originally infected in the gut or elsewhere.

Our estimate of the contribution of the gut to the plasma virus is also made independent of any estimate of the proportion of infected cells that reside in the gut. If infected cells in the gut produce virus at the same rate as infected cells elsewhere, then the proportion of infected cells residing in the gut will be the same as our estimated contribution of the gut to the plasma viral load. However if, for example, infected cells in the gut produced less virus than elsewhere, then the proportion of infected cells in the gut may be higher than would be expected based on their contribution to plasma viral load.

One limitation to our study is that only one site in gut has been sampled – rectal biopsy. Therefore, an important implicit assumption in this work is that the rectal tissue sampled is broadly reflective of the gut as a whole. Rectal biopsies represent only a subset of the entire gastrointestinal tract, and in fact differences have been reported by several groups, including ours, in terms of immunological features in different parts of the gastrointestinal tract [16]. However, other work has argued that similar levels of infection and depletion of CD4+ T cells are seen in the upper gastrointestinal tract and rectal biopsy [1,2]. This is a limitation that most studies of SIV/HIV infection of the gut have suffered from, as they have been limited by access to tissues, and by necessity have had to extrapolate from one or a few sites to the entire GALT. Rectal biopsies have been used in many studies of SIV immunology and pathogenesis [17-19] and, while perhaps not representative of the entire gastrointestinal tract immunology in every respect, we believe they provide robust and reproducible immunological and virological information in the context of SIV infection of macaques.

Another limitation of our study is that we can study the contribution of gut only in the period around the time of immune escape, when the proportion of WT virus differs between blood and gut. Our method should be generally applicable to studying the sources of viral production in HIV, as long as suitable markers for different viral strains can be identified. Further work should aim to clarify the contribution of gut to plasma virus at different times in HIV infection.

## Methods

The experimental protocol and primary data have been published elsewhere [12]. Briefly, 15 MamuA*01 rhesus macaques (10 previously vaccinated with 2 different vaccines and 5 unvaccinated) were challenged with SIV_mac239_. Blood, plasma, lymph nodes and rectal biopsies were collected at multiple times pre- and post-challenge. Plasma viral loads were determined using a real-time PCR assay specific for the SIV_mac239_ genome. 454 deep sequencing was used to identify and quantify escape mutations in MamuA*01-restricted immunodominant tat-SL8 epitope in multiple tissues. Escape at tat-SL8 was detected in all samples from all animals and occurred using a variety of amino acid substitutions.

### Ethics statement

These studies were carried out in strict accordance with the recommendations in the Guide for the Care and Use of Laboratory Animals of the National Institutes of Health, and were approved by the Emory University (AWA# A3180-01) and University of Pennsylvania (AWA# A3079-01) Institutional Animal Care and Use Committees. All animals were anesthetized prior to the performance of any procedure, and proper steps were taken to ensure the welfare and to minimize the suffering of all animals in these studies.

## Competing interests

The authors declare that they have no competing interests.

## Authors’ contributions

THV and GS conceived and designed the experiments, THV performed the experiments, GS contributed reagents and materials, JP did the statistical analysis and modeling, MPD conceived the project and all authors contributed to the writing of the paper. All authors read and approved the final manuscript.

## Supplementary Material

Additional file 1: Figure S1 Fraction WT in plasma and tissues on day 14. Click here for file

Additional file 2 Estimating the contribution of gut to total viral load. Click here for file

Additional file 3 Model of noncytolytic escape in two anatomical compartments. Click here for file

Additional file 4 Parameters and equations for different cases of suppression of production of WT virus in two compartments of different size. Click here for file
