# Supplementary material for: Estimating the contribution of the gut to plasma viral load in early SIV infection
Source: Retrovirology. 2013 Oct 14;10:105. doi: 10.1186/1742-4690-10-105 (PMC3854614; doi:10.1186/1742-4690-10-105)
Supplement: Additional file 1: Figure S1 — Fraction WT in plasma and tissues on day 14. [file 1742-4690-10-105-S1.pdf]

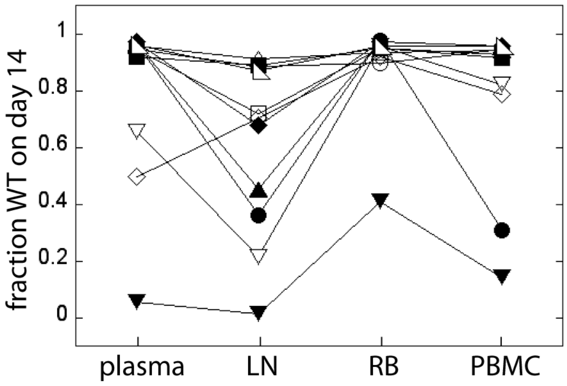

Figure S1. Fraction WT in plasma and tissues on day 14. There is little escape in plasma, rectal biopsy and PBMC, but there is some escape in lymph nodes preceding plasma, as observed in Vanderford et al. (2011).
