# Supplementary material for: Estimating the contribution of the gut to plasma viral load in early SIV infection
Source: Retrovirology. 2013 Oct 14;10:105. doi: 10.1186/1742-4690-10-105 (PMC3854614; doi:10.1186/1742-4690-10-105)
Supplement: Additional file 2 — Estimating the contribution of gut to total viral load. [file 1742-4690-10-105-S2.pdf]

A2. Estimating the contribution of gut to total viral load

If W is viral load of wild type (WT) and E is the viral load of escape mutants (EM) in plasma, then the fraction of WT in plasma is f_{PL},

$$f_{PL} = W / (W + E). \quad \text{Eq.A1}$$

The measured fraction of WT in the infected cells in rectal biopsy is f_{RB}^{cells},

$$f_{RB}^{cells} = I_{Wgut} / (I_{Wgut} + I_{Egut}), \quad \text{Eq.A2}$$

where I_{Wgut} and E_{Wgut} are cells infected by WT and EM virus in the gut, respectively. We define the fraction of WT in infected cells in all the other tissue compartments as f_{else}^{cells},

$$f_{else}^{cells} = I_{Welse} / (I_{Welse} + I_{Eelse}), \quad \text{Eq.A3}$$

where I_{Welse} and I_{Eelse} are infected cells in other compartments, respectively.

In estimating the contribution of each of the compartments to total viral load, we use the fact that half-life of free virus is much shorter than the half-life of infected cells, and that therefore the virus produced by infected cells is at any time proportional to the number of infected cells that produced it (quasistationary approximation).

The justification for the quasistationary approximation is as follows. There is a great heterogeneity in virus production rates at the individual infected cell level, and the production of virus by each infected cell can also vary over time. However, we assume that at each time point each anatomical compartment contains a large number (e.g. more than 1000) cells infected by different strains of virus, in various stages of their life cycle. We consider the wild-type quasispecies at tat-SL8 epitope as one group, and all other strains as the mutant group. At any point, some infected cells from each group may be bursting and some may have more moderate production rates, irrespective of the strain of virus that infected them. In making an estimate of the gut contribution to the plasma viral load, our assumption is that the *average* rate of production of virus *per cell* for each of the two groups in each compartment does not vary much over time. In this case, the number of infected cells from each of the two groups would be proportional to the amount of virus they produce (although the proportions of individual clones in each group could differ in individual infected cells and free virus over time).

If p_W and p_E are average virus production rates of cells infected by WT and EM, respectively, and c is the clearance of free virus, then the total viral load V_{gut} produced by infected cells in the gut would be:

$$V_{gut} = W_{gut} + E_{gut} = (p_W I_{Wgut} + p_E I_{Egut}) / c, \quad \text{Eq.A4}$$

and the total viral load V_{else} produced elsewhere would be

$$V_{else} = W_{else} + E_{else} = (p_W I_{Welse} + p_E I_{Eelse}) / c, \quad \text{Eq.A5}$$

where W_{gut} and E_{gut} are WT and EM virus produced in the gut, and W_{else} and E_{else} are WT and EM virus produced in other compartments, respectively.The fraction of WT in the *virus* produced by the infected cells in the gut (f_{gut}) and elsewhere (f_{else}) would then be respectively:

$$f_{gut} = \left[1 + \frac{p_E}{p_W}\left(\frac{1}{f_{RB}^{cells}} - 1\right)\right]^{-1} \quad \text{and} \quad f_{else} = \left[1 + \frac{p_E}{p_W}\left(\frac{1}{f_{else}^{cells}} - 1\right)\right]^{-1}. \qquad \text{Eq.A6}$$

The contribution C of the gut to the total viral load V in plasma is

$$C = V_{gut} / V = V_{gut} / (V_{gut} + V_{else}). \qquad \text{Eq.A7}$$

Using $W = W_{gut} + W_{else}$, WT viral load in plasma can be expressed as

$$W = f_{PL} V_{gut} / C = f_{gut} V_{gut} + f_{else}(1/c - 1)V_{gut}. \qquad \text{Eq.A8}$$

From Eq.A8 we obtain the contribution of the gut to total viral load in terms of fractions of WT in the virus produced by the gut and elsewhere:

$$C = \frac{f_{PL} - f_{else}}{f_{gut} - f_{else}}. \qquad \text{Eq.A9}$$

For given fractions of WT in plasma and in the virus produced by the gut, the maximum possible contribution of the gut would be if all the other tissues produced only EM virus and no WT at all. This corresponds to f_{else}=0:

$$C_{\max} = f_{PL} / f_{gut} = f_{PL}\left[1 + \frac{p_E}{p_W}\left(\frac{1}{f_{RB}^{cells}} - 1\right)\right] \qquad \text{Eq.A10}$$

a. WT and EMs are produced at the same average rate

If W_G and E_G are on average produced at the same rate ($p_W = p_E$), then from Eq.A6 the fraction of WT in the virus produced by the infected cells in the gut is the same as the fraction of WT in the cells themselves, so that:

$$C_{\max}^0 = f_{PL} / f_{RB}^{cells}. \qquad \text{Eq.A11}$$

as in Eq.1 in the manuscript. This assumption was used to estimate the contribution of virus produced in the gut to total viral load in Figure 2.

b. Error of estimate of the maximum gut contribution

If p_W and p_E are different, assuming that $p_W = p_E$ would create an error in our estimate of the maximum gut contribution. Instead of being given by Eq. A11, the maximum contribution would in fact be given by Eq.A10. The ratio

$$C_{\max} / C_{\max}^0 = f_{RB}^{cells} + p_E / p_W - p_E f_{RB}^{cells} / p_W \qquad \text{Eq.A12}$$

shows how many times would we over- or underestimate the gut contribution for a given measured value of f_{RB}^{cells}, depending on the actual ratio of p_E to p_W. The relative error in the estimate of the gut contribution to total viral load would increase linearly with p_E/p_W (Figure A2).

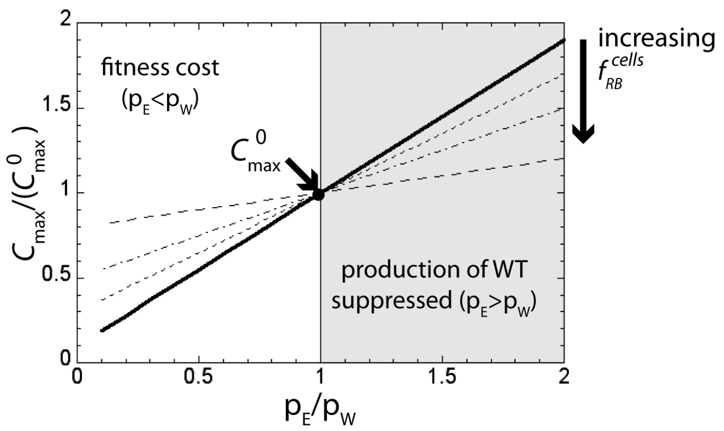

Figure S2. Relative error of estimate of the gut contribution for different fractions of WT in rectal biopsy, depending on the ratio of average production rates of EM and WT by infected cells.

If the mutations carry a fitness cost in being on average produced at a lower rate than WT by infected cells, then $p_W > p_E$. In this case the actual contribution $C_{\max}$ would be lower than the estimate $C^0_{\max}$ using $p_W = p_E$ (part of Figure A2 with white background).

It is also possible that, possibly due to some noncytolytic WT-specific immune response, cells infected with WT produce virus at a lower rate than cells infected with EM, so that $p_W < p_E$ (part of Figure A2 with grey background). In this case and we would be underestimating the maximal possible contribution of virus produced in the gut when using Eq.A11.

For all ratios of p_E/p_W the relative error is larger if the RB cells used for the estimate contain lower fraction of WT .
