# Supplementary material for: Estimating the contribution of the gut to plasma viral load in early SIV infection
Source: Retrovirology. 2013 Oct 14;10:105. doi: 10.1186/1742-4690-10-105 (PMC3854614; doi:10.1186/1742-4690-10-105)
Supplement: Additional file 3 — Model of noncytolytic escape in two anatomical compartments. [file 1742-4690-10-105-S3.pdf]

A3. Model of noncytolytic escape in two anatomical compartments

We use a simple model in which escape mutant quasispecies (E) competes with wild-type quasispecies (W) in two anatomical compartments of different sizes (large and small). The two compartments contribute to the total free virus that infects both compartments in the next round of infection. We are particularly interested in the case in which escape mutant overtakes wild type because WT-specific immune response suppresses production of WT virus by infected cells. Otherwise, WT and EM have the same susceptibility to infection and death rate of infected cells, but these parameters may differ between compartments. This is represented by the general system of equations:

Target cells:

$$dT_L/dt = -\beta_L T_L (W + E)$$
$$dT_S/dt = -\beta_S T_S (W + E) \qquad \text{Eq.A13}$$

Infected cells:

$$dI_{LW}/dt = \beta_L T_L W - \delta_L I_{LW}$$
$$dI_{LE}/dt = \beta_L T_L E - \delta_L I_{LE}$$
$$dI_{SW}/dt = \beta_S T_S W - \delta_S I_{SW} \qquad \text{Eq.A14}$$
$$dI_{SE}/dt = \beta_S T_S E - \delta_S I_{SE}$$

Free virus:

$$dW/dt = (p_{LW} I_{LW} + p_{SW} I_{SW}) - cW$$
$$dE/dt = (p_{LE} I_{LE} + p_{SE} I_{SE}) - cE \qquad \text{Eq.A15}$$

In Eq.A13-15, T_L and T_S are target cells for the virus in the large and small compartments respectively. The large compartment initially (at t=0) contains a much larger number of target cells:

$$T_L(t = 0) >> T_S(t = 0).$$

Susceptibilities to infection are β_L and β_S in the large and small compartments, respectively. Infected cells may generally die at different rates in different compartments: δ_L in the large compartment and δ_S in the small compartment. Free virus (W and E respectively) is produced from infected cells at rates that may depend on strain and compartment: p_{LW} and p_{SW} for wild-type virus produced in the large and the small compartment respectively, and p_{LE} and p_{SE} for escape mutant virus. Free virus clearance is assumed to be the same for all strains and in all tissues, and much faster than the death rate of infected cells. All parameters represent averages over time and over all cells from each group. Although the CD4 cells (target cells) are a heterogeneous lot, we assume that the average parameters are approximately constant.
