# Supplementary material for: Estimating the contribution of the gut to plasma viral load in early SIV infection
Source: Retrovirology. 2013 Oct 14;10:105. doi: 10.1186/1742-4690-10-105 (PMC3854614; doi:10.1186/1742-4690-10-105)
Supplement: Additional file 4 — Parameters and equations for different cases of suppression of production of WT virus in two compartments of different size. [file 1742-4690-10-105-S4.pdf]

## A4. Parameters and equations for different cases of suppression of production of WT virus in two compartments of different size

### a. Immune response is stronger in one compartment

Production rate of EM and all other parameters the same in both compartments: $p_{LE} = p_{SE} = p_E$; $\delta_L = \delta_S = \delta$; $\beta_L = \beta$.

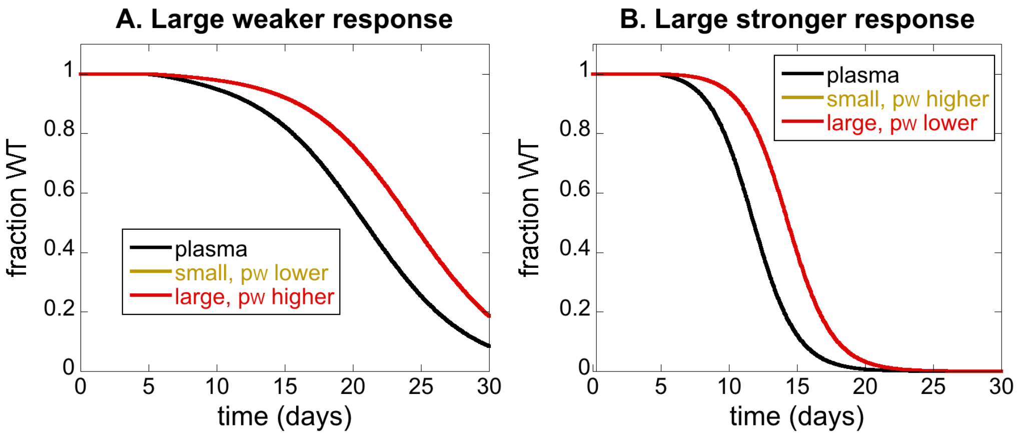


*Figure S3. Stronger suppression of WT in one compartment causes the same lag of escape in both compartments. A. Large compartment has weaker response; B. Larger compartment has stronger response. The difference in immune responses in compartments does not influence the relative lag of escape between tissues.*

| EQUATIONS | INITIAL CONDITIONS | |
|---|---|---|
| 1. Target cells<br>$dT_L/dt = -\beta T_L(W+E)$<br>$dT_S/dt = -\beta T_S(W+E)$<br><br>2. Infected cells<br>$dI_{LW}/dt = \beta T_L W - \delta I_{LW}$<br>$dI_{LE}/dt = \beta T_L E - \delta I_{LE}$<br>$dI_{SW}/dt = \beta T_S W - \delta I_{SW}$<br>$dI_{SE}/dt = \beta T_S E - \delta I_{SE}$<br><br>3. Plasma virus<br>$dW/dt = p_{LW} I_{LW} + p_{SW} I_{SW} - cW$<br>$dE/dt = p_E(I_{LE} + I_{SE}) - cE$ | $T_{0S}$ = 10 cells<br>$I_{0WS}$ = 0 cells<br>$I_{0ES}$ = 0 cells<br>$W_{0S}$ = 99.9 copies/ml<br>$E_{0S}$ = 0.1 copies/ml<br><br>PARAMETERS<br><br>$\beta = 2\times10^{-8}$ mL copies$^{-1}$ day$^{-1}$<br>$\delta = 0.8$ day$^{-1}$<br>$p_W = 5\times10^6$ copies/(ml·cell·day) first 5 days<br>$p_{Whi} = 2\times10^6$ copies/(ml·cell·day) later<br>$p_{Wlow} = 10^6$ copies/(ml·cell·day) later<br>$p_E = 5\times 10^6$ copies/(ml·cell·day)<br>$c = 20$ day$^{-1}$ | $T_{0L}$ = 1000 cells<br>$I_{0WL}$ = 0 cells<br>$I_{0EL}$ = 0 cells<br>$W_{0L}$ = 99.9 copies/ml<br>$E_{0L}$ = 0.1 copies/ml |

In the first days of infection we have $p_W = p_E$ in order to prevent escape from happening too early in infection. This mimics the situation where immune response develops during the first few days. WT production rates $p_{Whi}$ and $p_{Wlow}$ are first assigned so that $p_{Whi}$ is in the large and $p_{Wlow}$ is in the small compartment, and later the opposite.

**b. Susceptibility of cells to infection is higher in one compartment**

Production rates of WT ($p_W$) and EM ($p_E$) are different from each other, but are the same in both tissues. Infectivity $\beta_L$ in the large compartment is different from infectivity $\beta_S$ in the small compartment, but does not depend on viral strain. Death rate $\delta$ and virus clearance $c$ are strain- and compartment-independent.

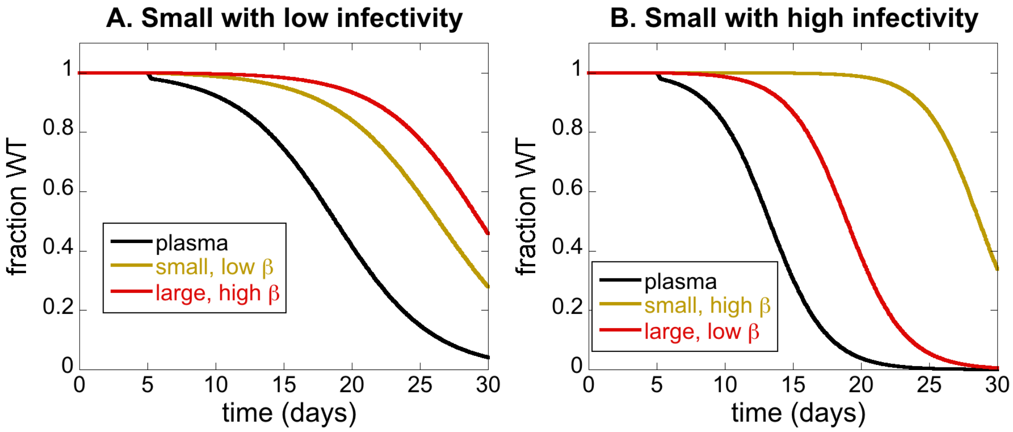


*Figure S4. If suppression of WT production is the same in all compartments, the lag of escape is larger in the compartment with higher infectivity. A. Smaller compartment has lower infectivity; B. Smaller compartment has higher infectivity.*

| EQUATIONS | INITIAL CONDITIONS | |
|---|---|---|
| 1. Target cells<br>$dT_L/dt = -\beta_L T_L (W+E)$<br>$dT_S/dt = -\beta_S T_S (W+E)$<br><br>2. Infected cells<br>$dI_{LW}/dt = \beta_L T_L W - \delta I_{LW}$<br>$dI_{LE}/dt = \beta_L T_L E - \delta I_{LE}$<br>$dI_{SW}/dt = \beta_S T_S W - \delta I_{SW}$<br>$dI_{SE}/dt = \beta_S T_S E - \delta I_{SE}$<br><br>3. Plasma virus<br>$dW/dt = p_W (I_{LW} + I_{SW}) - cW$<br>$dE/dt = p_E (I_{LE} + I_{SE}) - cE$ | $T_{0S}$ = 10 cells<br>$I_{0WS}$ = 0 cells<br>$I_{0ES}$ = 0 cells<br>$W_{0S}$ = 99.9 copies/ml<br>$E_{0S}$ = 0.1 copies/ml | $T_{0L}$ = 1000 cells<br>$I_{0WL}$ = 0 cells<br>$I_{0EL}$ = 0 cells<br>$W_{0L}$ = 99.9 copies/ml<br>$E_{0L}$ = 0.1 copies/ml |
| | PARAMETERS | |
| | $\beta_{hi} = 10^{-7}$ (large); $10^{-5}$ (small) mL copies$^{-1}$ day$^{-1}$<br>$\beta_{low} = 10^{-10}$ (small); $10^{-8}$ (large) mL copies$^{-1}$ day$^{-1}$<br>$\delta$ = 0.8 day$^{-1}$<br>$p_W = p_E = 10^6$ copies/(ml·cell·day) first 5 days<br>$p_W = 5\times10^4$ copies/(ml·cell·day) later<br>$c$ = 20 day$^{-1}$ | |

High and low susceptibilities to infection, $\beta_{hi} = 10^3\times\beta_{low}$, are different when the large compartment has $\beta_{hi}$ from when the large compartment has $\beta_{low}$, in order to make the peak viral load appear around 2$^{nd}$ week of infection.

### c. Death rate of infected cells is higher in one compartment

Production rates of WT ($p_W$) and EM ($p_E$) are different from each other, but are the same in both compartments. Death rate of infected cells $\delta_L$ in the large compartment is different from the death rate $\delta_S$ in the small compartment, but does not depend on viral strain. Infectivity $\beta$ and virus clearance $c$ are strain- and compartment-independent.

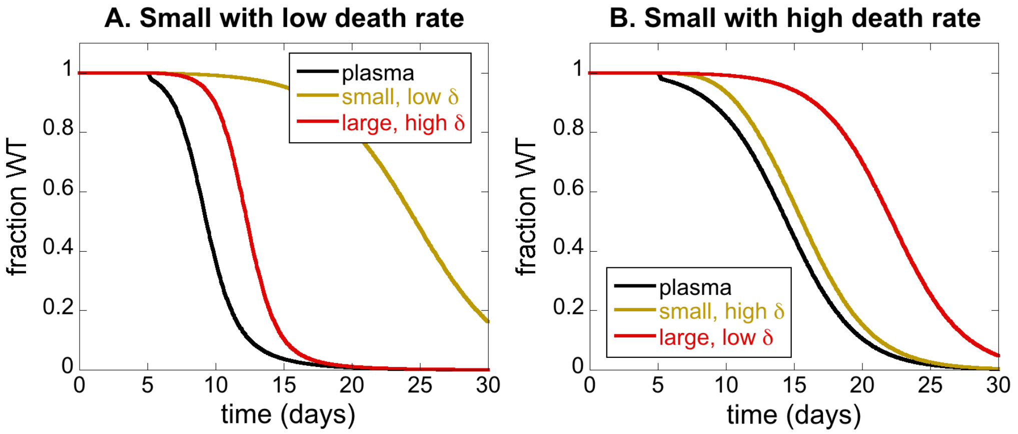


*Figure S5. With the same suppression of WT in all compartments, escape will lag the most in the compartment with the lowest death rate of infected cells. A. Small compartment has lower death rate; B. Small compartment has higher death rate*

<table>
<tr><th>EQUATIONS</th><th colspan="2">INITIAL CONDITIONS</th></tr>
<tr><td rowspan="3">1. Target cells<br>$dT_L/dt = -\beta T_L(W+E)$<br>$dT_S/dt = -\beta T_S(W+E)$<br><br>2. Infected cells<br>$dI_{LW}/dt = \beta T_L W - \delta_L I_{LW}$<br>$dI_{LE}/dt = \beta T_L E - \delta_L I_{LE}$<br>$dI_{SW}/dt = \beta T_S W - \delta_S I_{SW}$<br>$dI_{SE}/dt = \beta T_S E - \delta_S I_{SE}$<br><br>3. Plasma virus<br>$dW/dt = p_W(I_{LW} + I_{SW}) - cW$<br>$dE/dt = p_E(I_{LE} + I_{SE}) - cE$</td><td>$T_{0S}$ = 10 cells<br>$I_{0WS}$ = 0 cells<br>$I_{0ES}$ = 0 cells<br>$W_{0S}$ = 99.9 copies/ml<br>$E_{0S}$ = 0.1 copies/ml</td><td>$T_{0L}$ = 1000 cells<br>$I_{0WL}$ = 0 cells<br>$I_{0EL}$ = 0 cells<br>$W_{0L}$ = 99.9 copies/ml<br>$E_{0L}$ = 0.1 copies/ml</td></tr>
<tr><td colspan="2">PARAMETERS</td></tr>
<tr><td colspan="2">$\beta = 10^{-7}$ mL copies$^{-1}$ day$^{-1}$<br>$\delta_{hi}$ = 1.2 day$^{-1}$<br>$\delta_{low}$ = 0.5 day$^{-1}$<br>$p_W = p_E = 10^6$ copies/(ml·cell·day) first 5 days<br>$p_W = 5\times10^4$ copies/(ml·cell·day) later<br>$c$ = 20 day$^{-1}$</td></tr>
</table>

Death rates $\delta_{hi}$ and $\delta_{low}$ are first assigned so that $\delta_{hi}$ is in the large and $\delta_{low}$ is in the small compartment, and later the opposite.

### d. Conclusion

If escape were caused by preferential suppression of viral production by WT-infected cells, then we would expect escape in all compartments to lag after the escape in plasma. It would then be in principle possible for the larger compartment (contributing the major part of the plasma virus) to lag more after plasma than the smaller compartment, provided that it had much higher infectivity and/or much lower death rate of infected cells than the smaller compartment.

However, in this case it would not be possible to observe escape in any tissue preceding the escape in plasma, as is observed in some animals, for example:

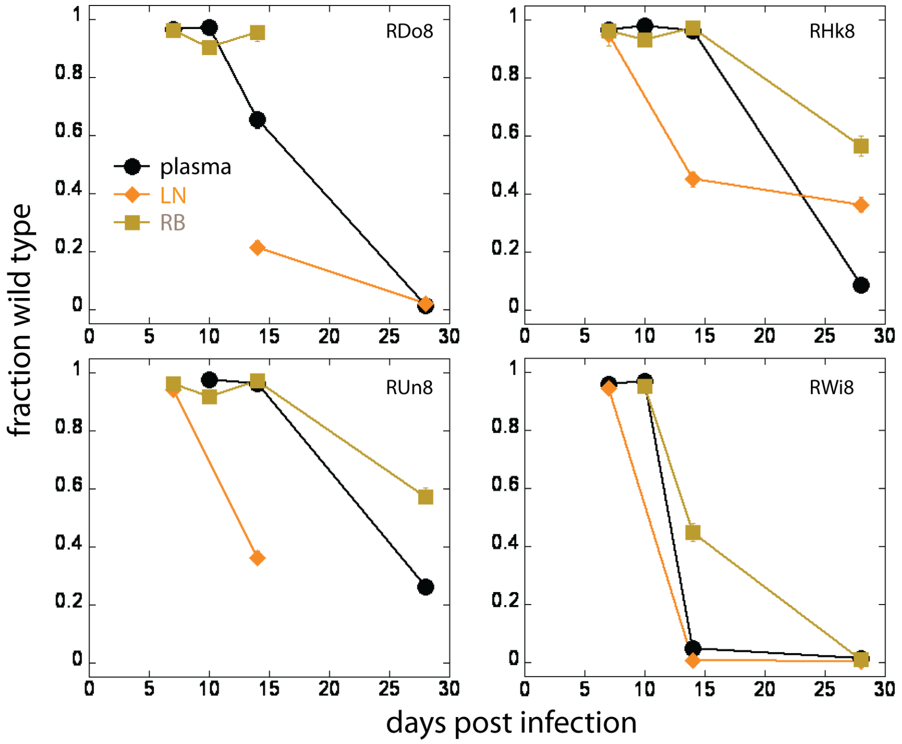


*Figure S6. Escape in lymph nodes precedes escape in plasma in some animals.*
